# Tailoring/Tuning Properties of Polyester Urea-Urethanes through Hybridization with Titania Obtained Using the Sol–Gel Process

**DOI:** 10.3390/polym15102299

**Published:** 2023-05-13

**Authors:** Dulce María González-García, Luis María Rodríguez-Lorenzo, Ángel Marcos-Fernández, Rodrigo Jiménez-Gallegos, Daniela Anahí Sánchez-Téllez, Lucía Téllez-Jurado

**Affiliations:** 1 Department of Metallurgy and Materials Engineering, ESIQIE, Instituto Politécnico Nacional, Mexico City 07738, Mexico; dgonzalezg@ipn.mx (D.M.G.-G.); rojimenezg@ipn.mx (R.J.-G.); danielatellez06@gmail.com (D.A.S.-T.); 2 Institute of Polymer Science and Technology (CSIC), Juan de la Cierva, 3, 28006 Madrid, Spain; luis.rodriguez-lorenzo@ictp.csic.es

**Keywords:** tailoring properties, polyester-urea-urethanes, hybrid materials, tissue engineering

## Abstract

Hybrid materials have been studied because in these materials the properties of organic components, such as elasticity and biodegradability, could be combined with the properties of inorganic components, such as good biological response, thereby transforming them into a single material with improved properties. In this work, Class I hybrid materials based on polyester-urea-urethanes and titania were obtained using the modified sol–gel method. This was corroborated using the FT-IR and Raman techniques which highlighted the formation of hydrogen bonds and the presence of Ti–OH groups in the hybrid materials. In addition, the mechanical and thermal properties and degradability were measured using techniques, such as Vickers hardness, TGA, DSC, and hydrolytic degradation; these properties could be tailored according to hybridization between both organic and inorganic components. The results show that Vickers hardness increased by 20% in hybrid materials as compared to polymers; also, the surface hydrophilicity increases in the hybrid materials, improving their cell viability. Furthermore, cytotoxicity in vitro test was carried out using osteoblast cells for intended biomedical applications and they showed non-cytotoxic behavior.

## 1. Introduction

Organic–inorganic hybrid materials may be more favorable than polymers or inorganic materials. The main advantage of organic–inorganic hybrids is that they are a single entity combining the different properties of both organic and inorganic components and as such they also present an opportunity to develop a large set of new materials with a wide spectrum of properties [1,2]. Moreover, hybridization between the organic and inorganic components offers incredible potential to create new materials with the properties needed in various advanced applications, such as tissue engineering [3].

Elastomers, such as polyurethanes (PUs), are polymer materials that exhibit good mechanical properties, tunable chemical properties, and cytocompatibility which are used in biomedical applications [4,5,6,7]. In polyester (urea-urethanes) polymers (PEUUs), the urea group shows stronger hydrogen bonds than the urethane group in polyurethanes, thus yielding better phase-separated morphology and exhibiting better mechanical properties. They are classified as a subclass of the generic polyurethane family. On the other hand, inorganic materials, i.e., ceramics and glasses, are stiff but fragile. However, ceramics, such as titania (TiO_2_), shows good interfacial properties with biological hosts, good corrosion resistance, lack of inflammatory response, and low cytotoxicity [8,9]. Hence, PEUUs–titania hybrids could be promising candidates in biomedical-related applications due to their great potential for tuning properties which can fit specific requirements.

The organic–inorganic hybrids based on polymers and titania have been proposed for different applications, such as PET covered with titania nanowires and nanorods for water pollutant removals with antibacterial activity [10] or self-cleaning textiles obtained using sol–gel coatings of silica and titania nanocomposites on polyester fibers with excellent behaviors [11]. Studies on TiO_2_ and polyethylene glycol (PEG) and the influence of the PEG molecular weight on flexibility and toughness in hybrid films was studied [12]. Studies have also been carried out on transparent aromatic polyamides (aramid)-based titania hybrid films prepared using the sol–gel process with improved elastic nature and photo-stability of the matrix [13]. Hybrids films of polyester-based polyurethane/titania using the sol–gel method were also reported [14] which found that introducing titania into the resin increases modulus, glass transition temperature (Tg), mechanical strength, abrasion resistance, hardness, and UV absorbance. Furthermore, polydimethylsiloxane (PDMS)-modified CaO–SiO_2_–TiO_2_ hybrids were successfully synthesized via a sol–gel process resulting in materials that offer high ductility, low elastic modulus, and high mechanical strength [15]. Hybrids based on poly caprolactones (PCL-d)/TiO_2_ obtained using a sol–gel process with varying proportion of the organic component have also been reported, which shows that an increase in PCL-d amounts improved cell viability [9]. Polyurethanes-TiO_2_ (PEU-TiO_2_) hybrids are barely found in the literature. In previous research, we reported the preparation of (PEU-TiO_2_) hybrids and showed that the addition of the inorganic component into the polyurethanes matrix improves the thermal and mechanical properties and promotes the bioactivity behavior compared to the polyurethane component [16]. However, such improvements have also been obtained in poly-ester-urea-urethanes (PEUUs) using an ester-lysine as a chain extender. This type of polymer showed higher stability in PBS than polyester-urethanes PEUs, due to the prominent hydrophobicity in their surfaces [17]. To date, no studies have been reported on hybrid materials obtained with polyester-urea-urethanes (PEUU) and titania, which have potential applications in tissue engineering. 

The aims of this work are to design hybrid materials based on polyester (urea-urethanes) and titania using a sol–gel process, evaluate the change in properties when this polymer is transformed into a hybrid material and analyze their potential use as biocompatible scaffolds for bone tissue regeneration.

## 2. Experimental Procedure

### 2.1. Synthesis

PEUUs/TiO_2_ hybrid materials were prepared in two steps: (1) synthesis of polyester (urea urethanes) (PEUUs), and (2) synthesis of organic–inorganic hybrids using a modified *sol–gel* method.

The starting reagents to prepare PEUUs were as follows: (1) ε-Caprolactone (ε-CL, Aldrich 97%); (2) 1,6-hexamethylene diisocyanate (HMDI, Aldrich 98%); (3) ʟ-Lysine-ethyl-ester-dihydrochloride (LYS, Aldrich 99%); (4) Triethylamine (TEA, Sigma Aldrich, St. Louis, MO, USA) and (5) N,N-Dimethylacetamide (DMac, Aldrich 99%). The starting reagents for the synthesis of hybrids were as follows: (1) Titanium (IV) butoxide (But-TiO_2_, Aldrich 97%); (2) hydrochloric acid (HCl, Fermont 37%); (3) isopropanol (IPA, Aldrich 99%); and (4) Tetrahydrofuran (THF, Aldrich) and deionized water. All reagents were purchased in Sigma Aldrich S.L (Madrid, Spain). The Polycaprolactone diol (PCL-d) of 1000 g/mol nominal weight was synthesized in the laboratory using the ring opening polymerization of the ε-Caprolactone following the procedure described in the literature [18].

#### 2.1.1. Synthesis of the Polyester (Urea-Urethanes) (PEUUs)

The polyester (urea-urethanes) (PEUUs) were synthesized using the previously prepared PCL-d as the soft segment; three different molecular weights (530, 1000, and 2000 umas) were assayed. The hexamethylene diisocyanate (HMDI) was used as the hard segment and ʟ-lysine-ethyl-ester-dihydrochloride (LYS) was used as the chain extender. The procedure is described in detail in a previous paper [17]. Briefly, the molar ratio of PCL-d:HMDI:LYS was adjusted to keep the weight of the hard segment at 30%. The synthesis was carried out through the two-stage polymerization technique. First, the polyester (PCL-d) reaction with the 1,6-hexamethylene diisocyanate (HMDI) was carried out in a three-neck flask under an inert atmosphere at 80 °C; these conditions were maintained for three hours to obtain the prepolymer containing the isocyanate groups. Second, the polymer (PEUUs) was obtained by adding the chain extender (LYS) and the triethylamine (TEA) in correct amounts, using N,N-Dimethylacetamide (DMac) as solvent at 80 °C for four hours. The added volume of solvent was adequate to achieve the dissolution of polyester (urea-urethanes) (PEUUs).

#### 2.1.2. Synthesis of the Hybrid Materials (THPs)

The PEUUs-based TiO_2_ hybrids (THPs) were obtained via a modified sol–gel method using the PEUUs previously synthesized. The synthesis of the hybrid materials was carried out via in situ reaction. First, the PEUUs were dissolved using tetrahydrofuran (THF) in a three-neck flask and then the appropriate amount of titanium (IV) butoxide (But-TiO_2_) was added to obtain a weight ratio of 80/20 of the organic/inorganic component. After that, water, HCl, and IPA (hydrolyzer, catalyzer, and solvent, respectively) were added, and the reaction was kept under constant stirring for one hour at 80 °C. The molar ratio of H_2_O:HCl:IPA was 3:0.3:4.5. The final hybrid materials were slowly dried at room temperature for several days. 

### 2.2. Characterization of the THPs 

Structural characterization of the (THPs) was achieved using Fourier Transform Infrared Spectroscopy (FT-IR), Raman Spectroscopy, and X-Ray Diffraction (XRD). FT-IR spectra were recorded via a Bruker spectrophotometer model Tensor 21 (FT-IR, Bruker, Ettlingen, Germany), equipped with an ATR (Attenuated Total Reflectance) PIKE model MIRacle, in transmittance mode using the mid-infrared region (from 400 to 400 cm^−1^) and 16 scans with a resolution of 4 cm^−1^. Samples were analyzed in the form of dried films. Raman spectra were obtained in a Renishaw in Via Reflex equipment with a laser diode of 785 nm coupled with a microscope and a Master Renishaw CCD camera (Raman, Renishaw plc, Wotton-under-Edge, UK). XRD analysis were carried out using a D8 FOCUS diffractometer (Bruker, Billerica, MA, USA) with a graphite filter of K𝛼Cu at 40 kV and 35 mA. The sweep in 2θ was set from 5° to 60°.

The thermogravimetric analysis (TGA) was carried out in a SETARAM brand calorimeter from 25 °C to 600 °C with a heating rate of 10 °C/min. Differential Scanning Calorimetry (DSC) analysis was conducted with a Perkin Elmer equipment from −80 °C to 200 °C and a heating rate of 10 °C/min. Two heating cycles were performed and the glass transition temperate (Tg) of each material was considered the second sweep. 

The morphology of the THPs was characterized using a high-resolution HITACHI SU 800 microscope with a voltage of 15 kV and a working distance of 8 mm to prevent degradation of the samples due to the presence of the organic component in the hybrid. 

Hydrophilicity property of the THPs was evaluated based on contact angle measurements obtained using a KSV Instruments LTD (CAM 200, KSV instrument Ltd., Helsinki, Finland) with an integrated camera CAM 200 employing the SFE CAM 2008 program. At room temperature, water was used as means of humidification.

The micro-hardness of the THPs was measured with a Leitz RZD-DO equipment (RZD-DO, Leitz, Oberkochen, Germany). The applied load was 4.809 N; the load holding time was 25 s at room temperature. The micro-hardness is reported in MPa.

### 2.3. Degradability Assay

The degradation of THPs was evaluated by immersing the samples in 20 mL of phosphate-buffered solution (PBS, pH = 7.4) at 37 ± 0.5 °C at intervals of 1, 3, 7, 14, 21, and 28 days. The pH value of PBS was registered at each test time. Additionally, the change in weight was recorded in samples dried at 37 °C at each test time. Weight loss was calculated as, %Weight loss = 100 − (W_2/(W_1)) × 100, where W_2 is the weight of the dried hybrid materials after being soaked at time t and W_1 is the initial weight of dried hybrids. Three replicas of samples were tested at each test time. 

### 2.4. Cytotoxicity Test

Cell viability of (THPs) was tested with osteoblast cell lines from (INNOPROT, P10979) during 24 h, using Termanox as a negative control. The MTT assay is a biochemical test used to assess the cytotoxicity of materials via qualitative measurement of cell viability and proliferation. The reagent used to produce stain is (3-[4,5-dimethyl-thiazol-2-yl]-2,5-diphenyl-tetrazolium-bromide)-sodium-succinate (MTT). The coloration changes from yellowish to blueish, suggesting reduction in the molecule by cellular mitochondrial dehydrogenase. Blue coloration is proportional to the number of living cells. Living cells can metabolize MTT, showing an indirect measure of cell viability. Three replicas of each hybrid material were tested.

#### Statistical Analysis of Cytotoxicity

The statistical analysis was carried out using STATA/SE software and Tukey’s post hoc test was analyzed for pairwise comparisons of means with equal variances. The statistical analysis of the cytotoxicity test was reported concerning the control, * *p* < 0.05.

## 3. Results 

Segmented polyester (urea-urethanes) were obtained via two-stage polymerization method using polycaprolactone diol (PCL-d) as the soft segment and both hexamethylen diisocyanate (HMDI) and ʟ-lysine-ethyl-ester-hydrochloride (LYS) as the hard segment.

Table 1 displays the codes and values for the contact angle and hardness of the PEUUs and hybrid materials (THPs). The molecular weight (Mw) and polydispersity (Mw/Mn) of PEUUs are also reported. As it can be seen in Table 1, the increase in the molecular weight of PEUUs directly influence the Vickers hardness values, due to the greater polymer chains formation and the low crystallinity, as discussed in the XRD section.

**Figure 1:**
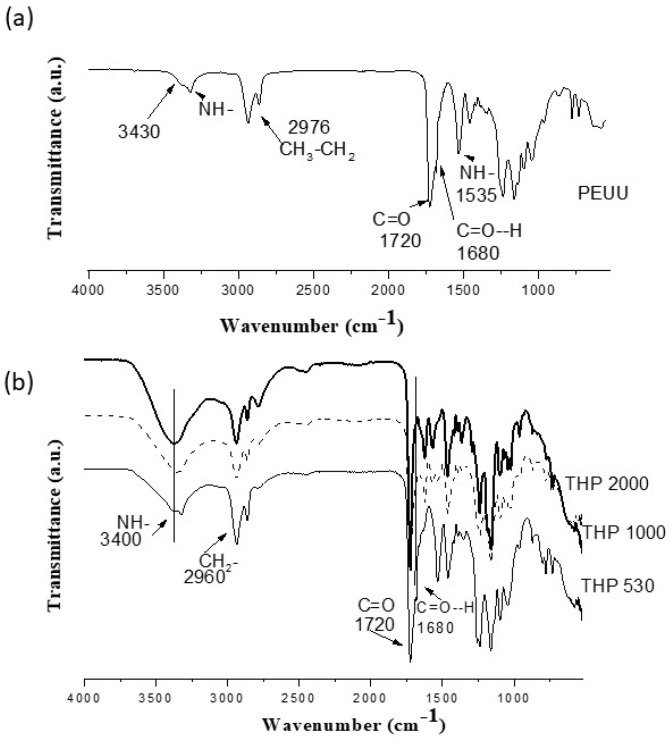
FT-IR spectra of the (**a**) PEUUs and (**b**) hybrid materials (THPs).

Figure 1a shows a representative FT-IR spectrum of the PEUUs. At 3320 cm^−1^, a broad band due to the vibrations of hydrogen-bonded urea and urethane groups in the N-H groups is observed. The shoulder located at 3430 cm^−1^ is due to the non-hydrogen bonded N–H groups related to the LYS, which hinders the molecular arrangement of the hard segment and promotes an asymmetric structure. The C=O absorption band located at 1720 cm^−1^ from the ester groups and non-hydrogen bonded carbonyl groups (C=O--H) at 1680 cm^−1^ are related to the PCL-d soft segment [19,20,21].

FT-IR spectra (Figure 1b) of the hybrid materials show the same bands as the PEUU spectrum described above. However, bands located at 3400 cm^−1^ and 1680 cm^−1^ due to the amine and carbonyl groups, respectively, are higher than those observed in PEUU. In addition, the broad bands located between 400 and 1000 cm^−1^ may also be attributed to the Ti–O–Ti and Ti–OH bonds of the inorganic components [22,23].

**Figure 2:**
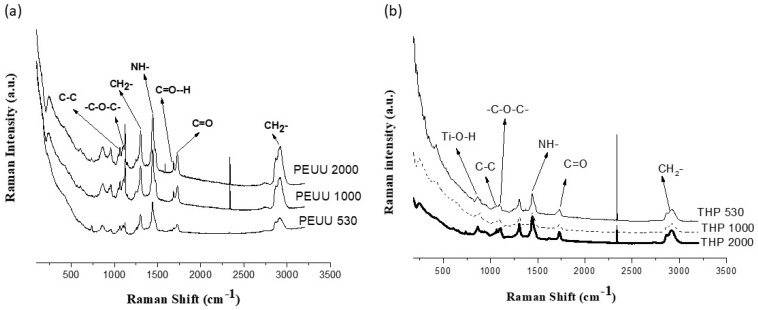
Raman spectra of the (**a**) PEUUs and (**b**) hybrid materials (THPs).

The Raman spectra of the PEUUs (Figure 2a) peaks at 2919 cm^−1^ and 1300 cm^−1^ due to the CH_2_- groups in the polyurethanes; peaks at 1738 cm^−1^ and 1676 cm^−1^ are due to the ester in both carbonyl groups (C=O) and non-hydrogen linked groups (C=O—H), respectively. Furthermore, peaks observed at 1450 cm^−1^, 1100 cm^−1^, and 1085 cm^−1^ are due to NH- groups, the C–O–C and C–C bonds, respectively [16,24]. The peaks at 240 cm^−1^ and 320 cm^−1^ are related to the polymer carbon skeleton.

On the other hand, the contribution of the inorganic component (TiO_2_) is appreciated in the Raman spectra of the hybrid materials. Figure 2b shows weaker Raman peaks related to the polymer matrix discussed before. However, some of them are shifted to a minor wavenumber, i.e., at 1070 cm^−1^ and 1095 cm^−1,^ due to the C–C and C–O–C groups, at 1440 cm^−1^ and 1730 cm^−1^ which is related to the NH- and C=O groups; also, the peak at 1676 cm^−1^ of (C=O–H) is no longer observed. The peak located at 860 cm^−1^ corresponds to the Ti–O–H groups [24]. These changes are related to the interaction between the organic and inorganic components. 

It is well known that the sol–gel process helps to obtain vitreous materials at temperatures as low as room temperature and a subsequent heat treatment is required for their crystallization [25]. According to reference [26], titania was obtained using sol–gel process with similar synthesis conditions as in this work (acid medium, solvent and hydrolysis agent/titanium alkoxide ratio). They obtained a crosslinked network of vitreous titania, which was then heat-treated to produce the crystalline phases, anatase and rutile. In the present research, titania was obtained by applying the sol–gel reaction in a polymeric matrix (PEUUs) showing a vitreous structure, which will be discussed later.

A study [27] on the crystalline titania spin-coated on the poly-carbonate surface reports that the controlling the pH values during the sol–gel synthesis in a basic medium, to obtain the amorphous titania and the upcoming curing temperature, influence its crystallization. In the analysis of Raman spectra, the authors found weak bands at 822 cm^−1^ and 922 cm^−1^ due to Ti–OH groups related to the non-crystalline titania. Additionally, they observed that the increase in the pH during the synthesis of the amorphous titania results in the increase in crystalline phases after curing temperature, showing signals in the Raman spectra at 150 cm^−1^ and 508 cm^−1^ (anatase phase) and at 442 cm^−1^ and 692 cm^−1^ (rutile phase). No evidence of these signals is seen in the Raman spectra in Figure 2b, indicating that the crystalline phases are not present in the hybrid materials of this work since no heat-treatment was conducted. These observations are based on the results of the XRD analysis, which will be analyzed below.

**Figure 3:**
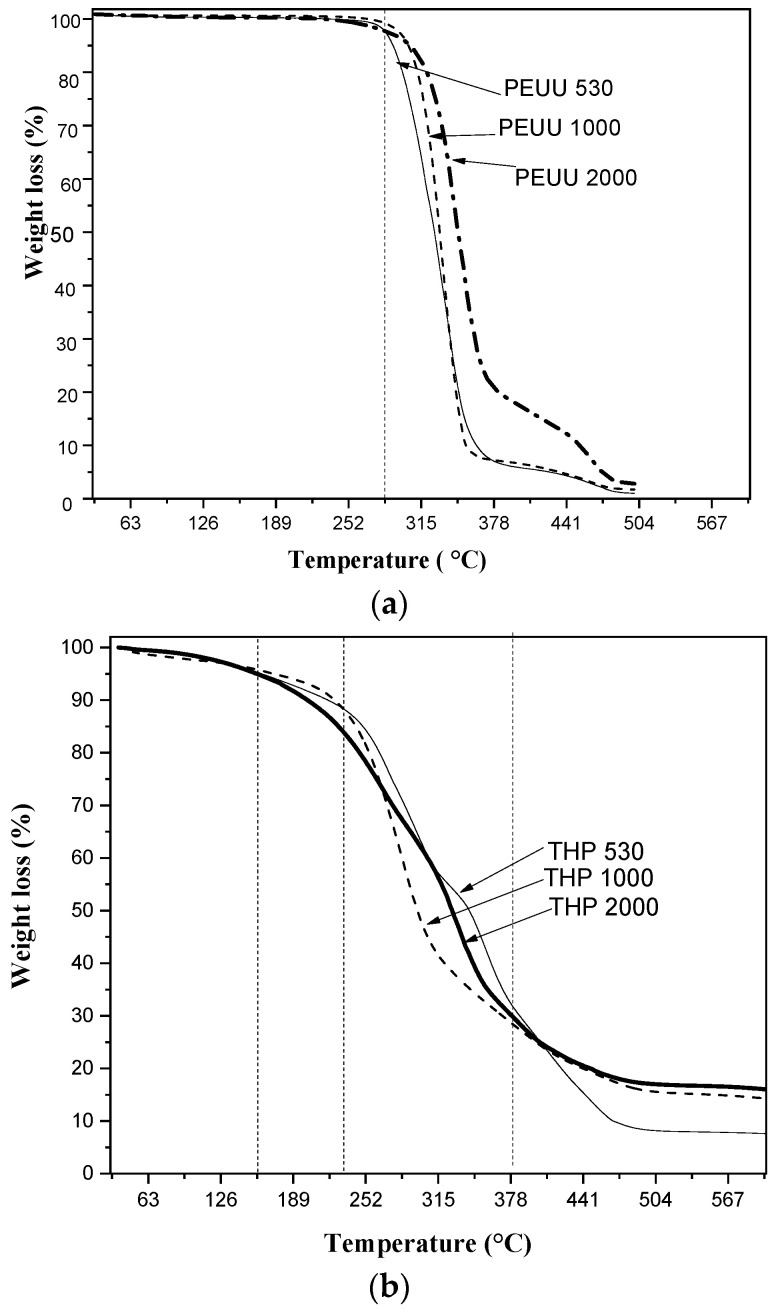
Thermograms of (**a**) PEUUs and (**b**) hybrid materials (THPs).

Figure 3 shows the thermogravimetric curves of the PEUUs and THPs. The thermogram of PEUUs (Figure 3a) shows a weight loss only at ~300 °C, attributed to polymer degradation. On the other hand, the thermograms of the THPs (Figure 3b) show three stages of weight loss. The first stage occurs below 100 °C with a weight loss of 5%, attributed to the loss of residual liquids (solvent and water from the polycondensation reactions) trapped in the hybrid network. The second stage occurs between 100 and 200 °C with a weight loss of ~20%, corresponding to the loss of the unreacted monomers and the polymer chains of low molecular weight. The third stage is related to the initial degradation of the polymeric component (PEUUs), which takes place above 200 °C showing a weight loss of ~55%. The complete decomposition of the polymer also is observed at ~ 500°C [28,29]. At the final thermal test (600 °C), the weight loss of both THP 1000 and THP_2000_ hybrid materials is ~80%, while the weight loss of THP_530_ hybrid is ~90%. As stated in the experimental section (Section 2.1.2), comprises a weight ratio of 80/20 of organic/inorganic components, hence, after the thermal test, the remaining weight corresponds to the inorganic component (TiO_2_) of the hybrid material. As can be seen, the remaining weight (20 wt.%) observed in THP_1000_ and THP_2000_ hybrids correspond to the weight of TiO_2_ added during the synthesis. However, THP_530_ remains at 10 wt.% (Figure 3b), indicating that not all TiO_2_ added were effectively incorporated into the organic matrix due to the different molecular weight of the polymeric precursors (PCL and PEUUs). The observed remaining weight of titania could be related to the higher repeat units of ester and urea-urethane groups in the polymer, which leads to a higher interaction of the hydrogen bonds with the inorganic component. Therefore, THP_1000_ and THP_2000_ (with higher molecular weight) have a higher interaction with titania since a greater quantity of polar groups from polymeric matrix interacts with the inorganic component in the hybrid materials [16]. On the other hand, THP_530_ has less polar groups available to interact, and consequently, loses part of the titania added during the synthesis, as can be seen in the thermogram in Figure 3b.

**Figure 4:**
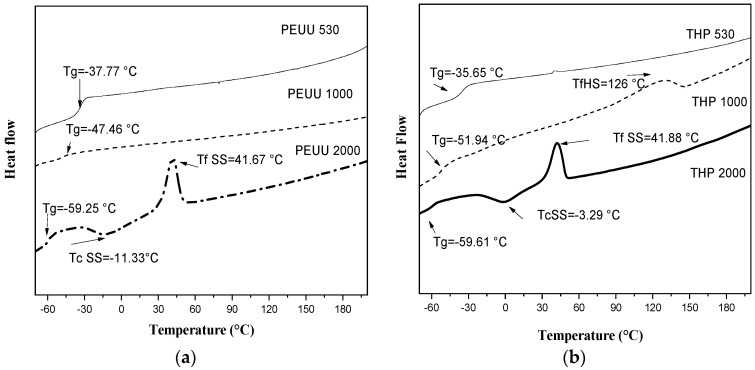
DSC of (**a**) PEUUs and (**b**) Hybrid materials (THPs).

The thermal transition of the PEUUs and THPs are shown in Figure 4. In addition, Table 2 summarizes the thermal transitions of the hybrids (THPs).

As mentioned above, the polyester-urea urethanes (PEUUs) are the polymeric component of the hybrid materials which is composed of soft (PCL of different molecular weight) and hard segment (diisocyanate and lysine). These segments could present a crystalline or amorphous structure [30]. As it is observed in Figure 4a, PEUU_530_, PEUU_1000_ and PEUU_2000_ show a glass transition temperature (Tg) at −37.77 °C, −47.46 °C, and −59.25 °C, respectively, and it is related to the molecular weight of the PEUUs. No melting temperatures are observed for PEUU_530_ and PEUU_1000_, but PEUU_2000_ shows a crystallization temperature at −11.33 °C due to the soft segment (Tc SS) and then the melting temperature is observed at 41.67 °C (Tf SS). The crystallinity in the soft segment of PEUU_2000_ is related to the major crystallinity in the PCL precursor with high molecular weight [31]. Furthermore, no thermal transitions were observed in the hard segment of this polymer. It could be explained in two ways: (1) the domains of the soft and hard segment were mixed in the first heating run, resulting in a single phase or, (2) the polymer showed a separation of the phases, one with rich domains in soft segment and other with rich domains in hard segment, as is discussed in other work [17].

On the other hand, Figure 4b shows the glass transition temperature at −35.65 °C, −51.94 °C, and −59.61 °C of the THP_530_, THP_1000_, and THP_2000_ hybrid materials, respectively. Since the hybrid materials are composed of 80 wt.% of the polymer, similar thermal transitions of PEEUs (Figure 4a) are expected. As can be observed, Tg value decreases as the molecular weight of the polymer increases. The THP_1000_ hybrid shows a melting temperature at 126 °C due to the hard segment (Tf HS), although this transition was not observed in its polymer precursor (PEUU_1000_). This could be attributed to the influence of the vitreous titania in the polymer matrix in which the titania interacts via hydrogen bonds with the hard segment domains. Finally, the crystallization (Tc) and the melting (Tf) temperatures of the soft segment in THP_2000_ take place at −3.29 °C and 41.88 °C, respectively, corresponding to the thermal behavior of the polymer precursor (PEUU_2000_) in the hybrid material. As can be seen, the hybrid materials showed an increase in Tg and a slight increase in Tf values compared to the PEUUs precursors. It could be related to the inorganic components that interacts principally with polar groups (carbonyl and amine groups) via hydrogen bonds, as discussed when FT-IR (Figure 1) and Raman (Figure 2) spectroscopy were conducted. This interaction promotes a different rearrangement in the structure of the polymer chains [32]. Therefore, the thermal behavior could be improved with the presence of an inorganic phase.

**Figure 5:**
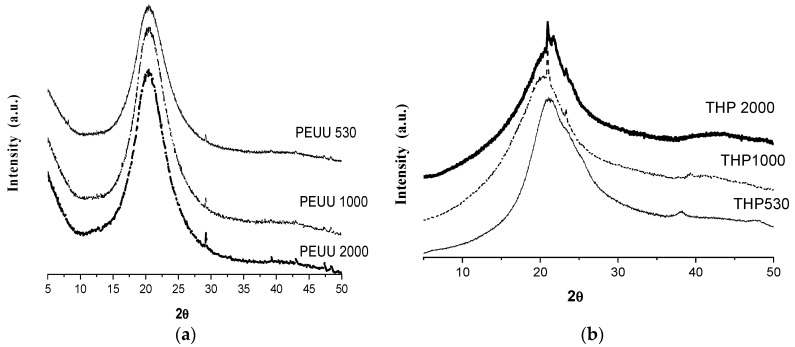
X-ray diffraction patterns of (**a**) PEUUs and (**b**) THPs.

The X-ray diffractograms of PEUUs and hybrid materials are shown in Figure 5.

The XRD patterns of PEUUs in Figure 5a show an amorphous halo with a maximum at ~22° in 2 theta, characteristic of low order arrangement materials. On the other hand, the XRD patterns of hybrid materials (Figure 5b) show a narrower amorphous halo, as described before, but a very low intensity peak at ~22° in 2 theta and a very small and narrow peak at ~38° in 2 theta is also observed. This could be related to the low order arrange of vitreous titania.

The lack of crystallinity diffraction peaks related to the titania crystalline phases (rutile at 27.4°, 36.07°, 39.17°, and 41.20°and for anatase at 25.27°, 36.93°, 37.78°, and 38.56° in 2 theta) [33] is due to the presence of vitreous titania, as discussed in the FT-IR and Raman sections. Other authors have also reported obtaining a vitreous titania using the sol–gel method with similar synthesis conditions reported in this work; they also studied the phase transformations of the vitreous titania to anatase and rutile phases by heat treatment [26].

**Figure 6:**
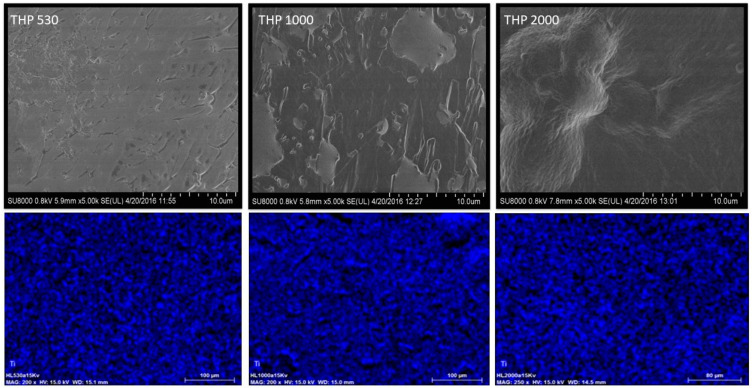
Morphology of the hybrid materials obtained using SEM and elemental Ti distribution from EDX mapping.

The SEM images of the morphology and the elemental mapping of Ti obtained using the EDX analysis of the hybrid materials are shown in Figure 6. As can be seen, the THP_530_, THP_1000_, and THP_2000_ hybrid samples are wrinkled and dense solids with some agglomerates on their surface. Additionally, it is observed that TiO_2_ is well-dispersed on the hybrid surfaces.

**Figure 7:**
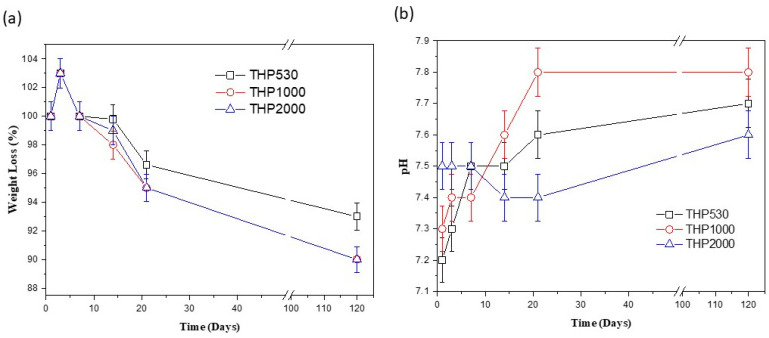
(**a**) Change in weight of THPs after being immersed in PBS and (**b**) pH variation of PBS at each time of test. Physiological conditions were used.

On the other hand, the analysis of the degradability test was carried out by means of the changes in weight of the hybrid samples after being immersed in the buffer solution (PBS), and the variation of the pH values of this solution at different test times are shown in Figure 7. 

The tendency towards weight loss exhibited by the hybrid materials is similar in all the three cases. On the first day, the weight of the hybrids increases due to the absorption of the PBS solution, and on the third day, they experience a gradual weight loss. The total weight loss of the three hybrids is ~10% at 120 days of testing.

The pH values observed in the first three days of testing are related to the release of residual reagents and solvents trapped in the hybrid network. After this time, the pH shows a value of 7.4–7.5, which remains until 14 days of testing. Then, the pH value increases slightly without exceeding a pH value of 7.8 due to the amine groups of the hybrids released into the solution. This fact shows a low degradation of hybrid materials.

**Figure 8:**
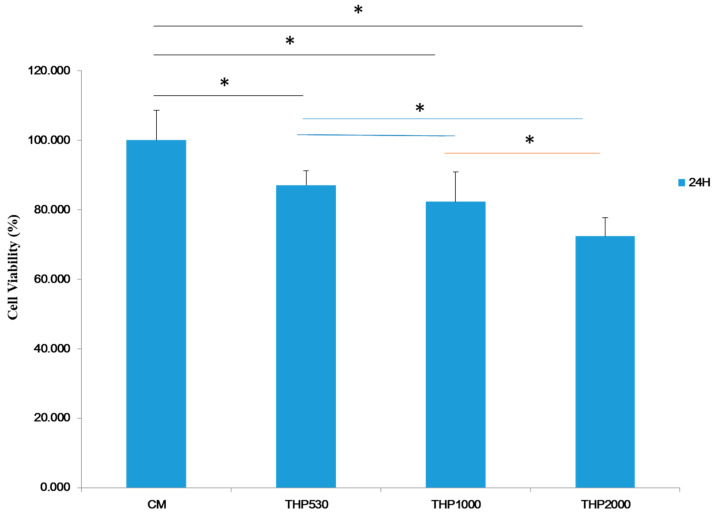
Cell viability of the hybrid materials at 24 h using an osteoblastic cell line. One-way ANOVA (*) indicates statistical difference from the control *p* < 0.05.

The results of the cell viability (CV) assay for the three hybrid materials and for the control (TERMANOX) at 24 h are shown in Figure 8. The THP_530_ and THP_1000_ hybrid materials present greater cell viability (≥80%) than the THP_2000_ hybrid (≥70%). However, the observed cell viability values indicate that the hybrids are non-cytotoxic materials, as mentioned in other works with similar values of cell viability [34,35]. The lower cell viability of THP_2000_ could be related to the amount of residual solvent trapped in the hybrid. However, it is assumed that after a few days, the residuals will be eliminated, according to the results of the degradation test. Furthermore, cell proliferation begins on the first day of the in vitro test.

## 4. Discussion

In a previous work [17], the effect of the chemical nature of both the hard segment and the soft segment of polyurea-urethanes on the chemical, mechanical and biological properties of polymers was analyzed. The obtained polyurea-urethanes showed good mechanical behavior and excellent biostability. Furthermore, in the present work, the hybridization of these polymers with titania and synthetization of the THPs via the sol–gel process was demonstrated using FT-IR and RAMAN analysis wherein the non-hydrogen bonded urea groups decrease, and the linked carbonyl groups increases. This could be related to the new hydrogen bonds formed between the amine and carbonyl groups from the polymer and the inorganic component. Such interactions between organic and inorganic component were thought to be responsible for forming Class I hybrids, as described in the literature [36,37]. In addition, the contribution of titania is noted in the Raman peak at 860 cm^−1^ due to Ti–O–H bonds. The interactions between inorganic and organic components in the hybrid directly influence important properties of the materials, such as thermal and mechanical properties.

The Vickers hardness values (Table 1) of PEUU 530, PEUU_1000_ and PEUU_2000_ are 8, 35 and 48 MPa, respectively, while the Vickers hardness values of hybrid materials are 10, 50 y 60 MPa for THP_530_, THP_1000_, and THP_2000_, respectively, showing values 20% higher than the respective PEUUs. This increment is attributed to the presence of the vitreous titania which is well dispersed into the polymer matrix of the hybrids. Additionally, the hardness of polymers and hybrid materials depends on the molecular weight of their respective precursor: as the molecular weight of the precursor increases, the hardness also increases in both polymers and hybrid materials.

Hydrophilicity is a desirable property for materials interacting with cells for tissue regeneration, as shown in reference [38], in which the authors reported that an increment in the contact angle increases cell adhesion and proliferation.

The degradation in an aqueous medium and the release of non-toxic products are necessary properties of biomaterials intended for tissue regeneration applications. In the present work, the hybrid materials show a weight loss of ~10% after being immersed in PBS for 120 days.

The introduction of the inorganic phase (TiO_2_) in the polymeric matrix through sol–gel reactions improve the mechanical, thermal, and biological properties of the hybrid materials. Additionally, titania is non-cytotoxic and is well known to promote osteoconductivity [16,39,40], as seen in the cell viability test using the human osteoblast cell line presented in Figure 8.

Additionally, the inorganic phase can be introduced as particles or synthesized in situ with the organic phase in composite and hybrid materials providing the possibility to tailor the mechanical and physicochemical properties of materials for different applications. Related works [41,42,43] reported materials based on polyurethanes with the addition of particles or in situ synthesis of titania in different concentrations to improve the properties in the final materials for various applications. In reference [44], the authors synthesized in situ composite materials based on polyurethanes and commercial TiO_2_ particles. They mentioned that TiO_2_ interacts with the hydrogen from carbonyl groups of the polymer and analyzed its different properties. They found that the thermal and mechanical properties improved with the introduction of TiO_2_.

In our work, hybrid materials were obtained through the in situ reaction of vitreous TiO_2_ and PEUU using the sol–gel process. For this purpose, hydrolysis–condensation reactions were carried out within the polymer, which resulted in a strong interaction between the organic and inorganic phases, providing materials with better thermal and mechanical behavior and promising biological results.

## 5. Conclusions

A novel material based on polyester (urea-urethanes) and titania was synthesized using the sol–gel reaction. The strong interaction between the organic and inorganic phases influences thermal and mechanical properties. Additionally, the surface of the materials is more hydrophilic, and the in vitro cytotoxicity of hybrid residues was analyzed at 24 h, which showed that they were non-cytotoxic to human osteoblast cells.

## Figures and Tables

**Table 1:** Sample codes, contact angle and hardness of PEUUs and hybrid materials (THPs), and Mw and Mw/Mn of PEUUs.

Sample Codes of PEUUs	PEUUs Mw (g/mol)	PEUUs Mw/Mn	Contact Angle	Vickers Hardness (MPa)	Sample Codes of Hybrids	Contact Angle	Vickers Hardness (MPa)
PEUU_530_	27,600	1.2	70°	8	THP_530_	48°	10
PEUU_1000_	136,700	1.8	73°	35	THP_1000_	45°	50
PEUU_2000_	196,300	2.0	77°	48	THP_2000_	40°	60

**Table 2:** Thermal transitions of THPs.

Hybrid	Heat Sweep (−80–180 °C)			
	Tc * SS	Tf * SS	Tf * HS	Tg
THP_530_	-----	-----	-----	−35.65
THP_1000_	-----	-----	126	−51.94
THP_2000_	−3.29	41.88	-----	−59.61

* SS = Soft segment. * HS = Hard segment.

## Data Availability

The data that support the findings of this study are available on request from the corresponding author.
